# Post-treatment surveillance imaging in head and neck cancer: a systematic review

**DOI:** 10.1186/s13244-023-01578-4

**Published:** 2024-02-05

**Authors:** Stefaan Van Hoe, Robert Hermans

**Affiliations:** 1 Faculty of Medicine, KU Leuven, Leuven, Belgium; 2 Department of Radiology, University Hospitals Leuven, Herestraat 49, Leuven, 3000 Belgium; 3 Department of Imaging and Pathology, KU Leuven-University of Leuven, Leuven, Belgium

**Keywords:** Head and neck cancer, Post-treatment surveillance, Squamous cell carcinoma, Systematic review, Neoplasm recurrence

## Abstract

**Background:**

In patients treated for head and neck cancer, imaging studies are usually obtained within 3–6 months after treatment for assessment of treatment response. After 6 months, most guidelines advocate clinical follow-up, with imaging reserved for patients with clinically suspect or equivocal findings. However, some guidelines do recommend systematic imaging surveillance, and many clinicians tend to include some type of imaging in their follow-up schemes.

**Objectives:**

This systematic review focuses on the usefulness of routine (systematic) post-treatment imaging surveillance of head and neck cancer beyond the first 3–6-month baseline imaging study.

**Methods:**

A systematic literature search was conducted using PubMed and Google Scholar. Additional studies were identified by reviewing reference lists. Only original studies and review papers were considered. Results obtained with systematic post-treatment surveillance imaging were compared to symptom-directed imaging and/or clinical finding-directed imaging.

**Results:**

Five hundred twenty-one records were identified through the database search, and 44 additional records were identified through other sources. Forty-eight articles were selected for the final review.

Analysis of these records showed that almost half of cases of locoregional recurrences and/or metastases were only detected by imaging (40.9%), and the mean time of detection of recurrent or metastatic disease (11.5 months) was well beyond the period of the first post-treatment scan. Most authors reported superior results with PET-CT when compared to other imaging techniques.

**Conclusion:**

Strong arguments were found in favor of systematic imaging surveillance in locoregional advanced head and neck cancer during at least one and preferably 2 years after treatment.

**Critical relevance statement:**

Analysis of the selected records showed that almost half of cases of locoregional recurrences and/or metastases were only detected by imaging. This systematic review suggests that imaging may currently be underused in the post-treatment surveillance of patients with head and neck cancer.

**Key points:**

• This systematic review focuses on the usefulness of long-term systematic imaging surveillance in patients treated for head and neck cancer.

• Analysis of 521 articles revealed that systematic imaging allowed the initial detection of locoregional recurrences and/or metastases in more than 40% of patients.

• Imaging may currently be underused in the post-treatment surveillance of patients with advanced head and neck cancer.

**Graphical Abstract:**

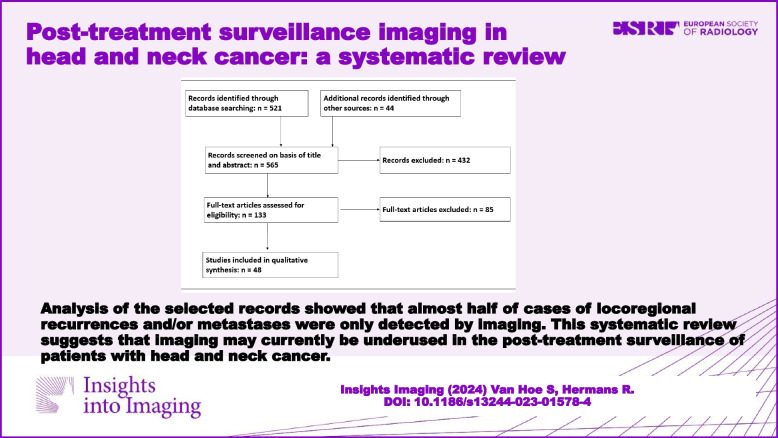

**Supplementary Information:**

The online version contains supplementary material available at 10.1186/s13244-023-01578-4.

## Introduction

Worldwide head and neck cancer accounts for approximately 900,000 cases and over 400,000 deaths annually [1]. Most malignant tumors are head and neck squamous cell carcinomas (HNSCC). Treatment options include surgery, radiation therapy, and chemotherapy, or a combination of these modalities.

Approximately 50% of patients with locally advanced head and neck cancer will develop a recurrence [2, 3]. Distant metastases are present in about 10% of cases at the time of diagnosis, with an additional 20–30% developing during the course of the disease [4]. Up to 80–85% of metastases from HNSCC are observed in the lungs. Bone metastases account for 15–39%, while the liver is a affected in 10–30% of cases.

Patients treated for primary HNSCC also have a high rate (3 to 5% per year) of developing second primary tumors [5, 6].

After treatment, there are two principal periods of surveillance: immediate posttreatment (within 6 months) and long-term (6 months onward).

There is now a consensus that, for patients with locoregionally advanced disease (e.g., T3-T4 primary or N1 + nodal staging), imaging should be performed within 3 months to 6 months after completion of definitive treatment in order to assess treatment response [7–9].

On the other hand, the utility of long-term imaging surveillance is subject of debate. Currently, controlled prospective data demonstrating a survival benefit for any follow-up strategy in this specific context do not exist. While some guidelines recommend systematic imaging, others state that additional post-treatment imaging should be reserved for patients with worrisome or equivocal signs and/or symptoms [8–17]. The National Comprehensive Cancer Network (NCCN), for instance, does not recommend routine imaging for surveillance in asymptomatic patients, except for specific patient populations. These include patients with primary nasopharyngeal carcinoma, a history of smoking, or a primary tumor in areas inaccessible by clinical examination [8].

Surveys of practicing physicians have shown that many clinicians do not follow the NCCN guidelines and believe systematic imaging may be appropriate in at least some of their patients, despite lack of symptoms [18–20]. In other words, current guidelines are inconsistent and contradictory, and clinicians tend to develop their own strategy, based on personal and institutional preference.

This systematic review exclusively focuses on the usefulness of routine post-treatment imaging of head and neck cancer beyond the first 3–6-month baseline imaging study. Hereby, the strategy of systematic follow-up imaging is compared to symptom-directed imaging and/or clinical finding-directed imaging. This is an important issue because both overuse and underuse of imaging bears significant risk and disadvantages. Overuse of imaging may cause patient stress, unnecessary expenses [21] and iatrogenic side effects (e.g., related to radiation in CT or PET/CT). Underuse of imaging, on the other hand, may lead to missed opportunities for early detection of recurrences and salvage treatment.

In this study, we review the retrospective data and observational studies that are available. Possible outcomes studied are the detection of locoregional recurrence or metastases, change of therapeutic regimen, and survival. The focus is on squamous cell carcinoma of the pharynx, oral cavity, and larynx. Both qualitative and quantitative results are obtained, and the collected data are used to provide practical recommendations.

## Methods

### Study protocol and registration

Methods of the analysis and inclusion criteria were specified in advance and documented in a protocol accordingly to the PRISMA guidelines [22, 23].

### Eligibility criteria

Study eligibility criteria are given below according to the PICOS framework (populations, interventions, comparators, outcomes, and study designs of interest) as well as other study-specific elements.

1. Types of participants/populations (**P**): patients who underwent treatment for head and neck cancer, with curative intent. Further inclusion criteria:
  Squamous cell carcinoma
  Primary tumor in nasopharynx, oropharynx, hypopharynx, larynx, or oral cavity
  Patients treated with surgery, radiotherapy, and/or systemic therapy
  Patients with or without risk factors (smoking etc.)

(2) Types of intervention (**I**): systematic (planned) post-treatment surveillance imaging studies.
  This review does not cover the first post-treatment scan, typically performed after 3–6 months
  Included are studies on post-treatment imaging surveillance after the first post-treatment scan
  Techniques: computed tomography (CT), magnetic resonance imaging (MRI), positron emission tomography (PET), PET-CT, ultrasonography (US)
  Body locations studied: neck, chest, and/or full body
  Search for local recurrence, regional recurrence (e.g., lymph nodes in the neck), distant metastases

(3) Types of comparators (**C**): other methods used for detection of locoregional recurrence or metastases.
  Clinical exam
  Patient anamnesis (symptoms)
  Imaging studies requested based on suspect clinical exam of patient symptoms (in contrast to systematic—planned—imaging surveillance)
  Endoscopic studies
  Other types of imaging studies (e.g., PET-CT versus CT)
  Any other test

(4) Types of outcome measures (**O**):
  Detection of local recurrence, regional recurrence (e.g., metastatic lymph nodes), distant metastases
  Change in therapeutic regimen
  Impact on survival

(5) Types of studies (**S**):
  Included: original studies, abstracts of conference papers (if containing sufficiently detailed information), doctoral dissertations
  Excluded: letters, editorials, reviews, and case reports
  Studies can be prospective or retrospective. Studies using mathematical models (e.g., Markov models) were also included

(6) Other study-specific elements:
  Reports published before 2000 were not considered during the initial search
  Report language was restricted to English, French, and Dutch
  Only published reports were considered
  No restriction was put on the length of follow-up

### Information sources

PubMed and Google Scholar were used for database search. The PubMed and Google Scholar search was done between August 1 and September 27, 2022. The search was developed and conducted by SVH.

Supplementary approaches to identify studies are as follows: first, the reference lists of all review articles obtained via the database search described above were checked, and additional records fulfilling the eligibility criteria were identified. Next, all articles citing the records found via the database search described above were identified via Google Scholar. Next, the reference list of the article “Posttreatment surveillance of squamous cell carcinoma of the head and neck” in the electronic database “*UpToDate*” was checked. Finally, the reference lists of the articles obtained via the last two steps were scrutinized.

### Electronic database search strategy

The following searches were done:

- PUBMED SEARCH: ((head and neck cancer[Title/Abstract]) AND (surveillance[Title/Abstract])) AND (imaging[Title/Abstract])
- PUBMED SEARCH: ((head and neck cancer[Title/Abstract]) AND (surveillance[Title/Abstract])) AND (posttreatment[Title/Abstract])
- References in NCCN guidelines
- PUBMED SEARCH: ((head and neck cancer[Title/Abstract]) AND (follow-up[Title/Abstract])) AND (posttreatment[Title/Abstract])
- PUBMED SEARCH: ((head and neck cancer[Title/Abstract]) AND (surveillance[Title/Abstract])) AND (tomography[Title/Abstract])
- PUBMED SEARCH: (((cancer[Title/Abstract]) AND (surveillance[Title/Abstract])) AND (imaging[Title/Abstract])) AND (pharynx)
- PUBMED SEARCH: (((cancer[Title/Abstract]) AND (surveillance[Title/Abstract])) AND (imaging[Title/Abstract])) AND (oral cavity[Title/Abstract])
- PUBMED SEARCH: (((cancer[Title/Abstract]) AND (surveillance[Title/Abstract])) AND (imaging[Title/Abstract])) AND (larynx[Title/Abstract])
- GOOGLE SCHOLAR SEARCH: “head and neck cancer posttreatment imaging surveillance”
- GOOGLE SCHOLAR SEARCH: “head and neck cancer posttreatment imaging follow-up”
- GOOGLE SCHOLAR SEARCH: “pharynx cancer imaging surveillance”
- GOOGLE SCHOLAR SEARCH: “oral cavity cancer imaging surveillance”:
- GOOGLE SCHOLAR SEARCH: “larynx cancer imaging follow-up surveillance”:
- GOOGLE SCHOLAR SEARCH: “head and neck cancer tomography surveillance”:
- GOOGLE SCHOLAR SEARCH: all articles citing the records found via the database search described above were identified via Google Scholar (“cited by”).
- UPTODATE: the reference list of the article “Posttreatment surveillance of squamous cell carcinoma of the head and neck” [24] in the electronic database “UpToDate” was checked.

### Study selection

Eligibility assessment was performed by one author (S.V.H.) and reviewed by a second author (R.H.). Records obviously not covering the topic of the review were excluded from further consideration. Disagreements between reviewers were resolved by consensus.

Records obtained from the PubMed and Google Scholar searches were compared, and duplicates were removed. Records obtained via the supplementary approaches to identify studies (see above) were only retained if the record was not yet available in the list of records obtained via database search (i.e., duplicates were removed on a record-by-record basis).

All records obtained via the database search and via the supplementary approaches were screened based on evaluation of title and abstract. Inclusion and exclusion criteria are described above in accordance with the PICOS framework.

**Fig. 1:**
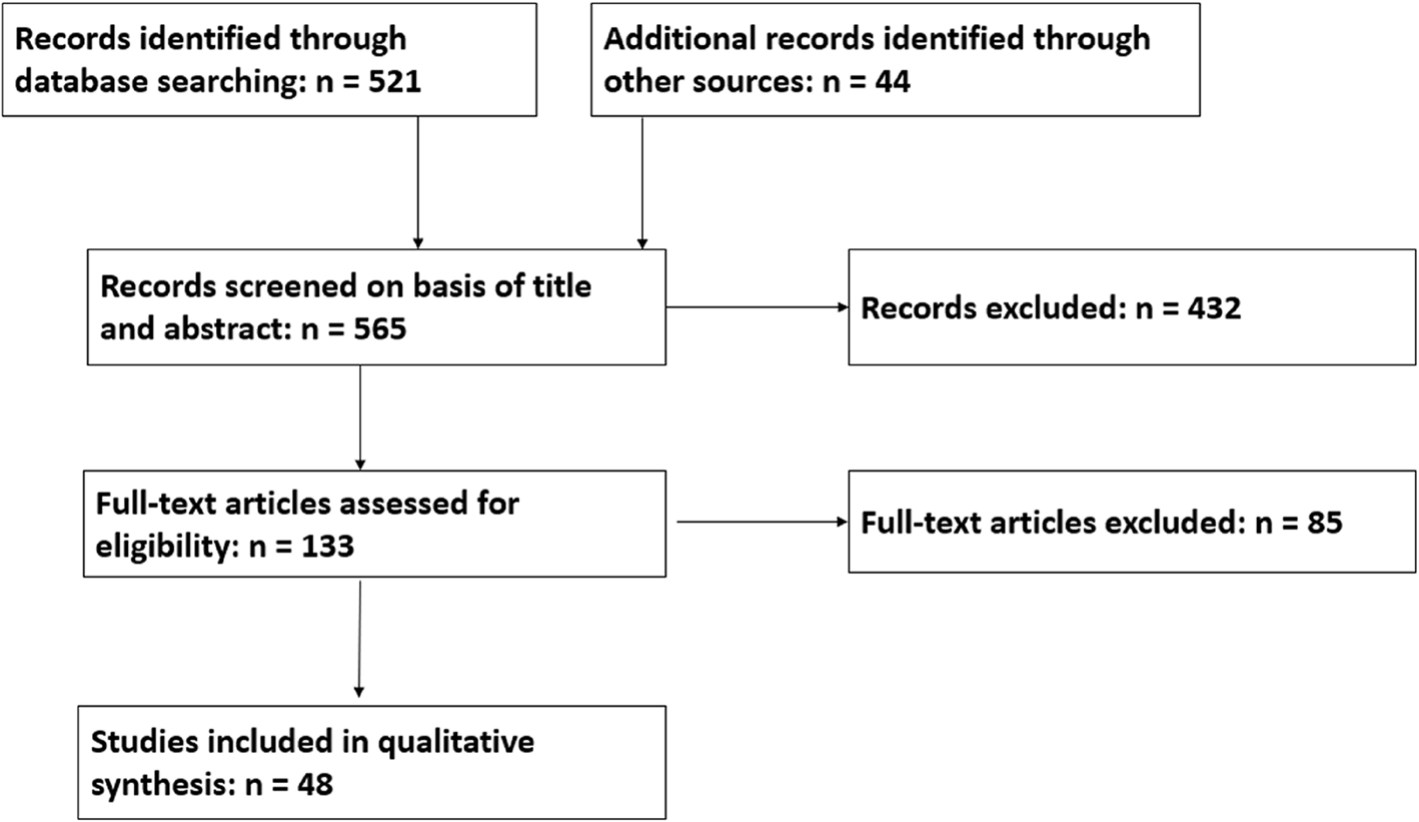
PRISMA flowchart

The potentially eligible records not excluded during this step were promoted to the next stage of the review: full text screening (see Fig. 1). All articles selected for full text review were read by one author (S.V.H.). This work was reviewed by RH and discrepancies were resolved in consensus.

During all steps in the process, special attention was given to the removal of duplicate studies.

The details of each article (author, title, publication year) were recorded in an excel file. In the file, each article was assigned to one of the following categories, in accordance with the methodology described above:

- Category 1: Record obtained via database search or via the supplementary approaches but excluded based on evaluation of title and abstract
- Category 2: Records considered eligible based on review of title and abstract but excluded after full text review
- Category 3: Records included after full text review

An overview of all articles, together with their category, is given in the electronic supplementary material (Manuscript selection sheet).

In addition to the reasons given above, reasons to exclude articles were as follows:

- Missing details in the materials and methods or results section, making interpretation or data extraction difficult or impossible
- Full text article not accessible
- Different research topic than anticipated based on reading the title and abstract
- No comparison between systematic (routine) surveillance imaging and another type of surveillance (presentation of results obtained with one method, without any comparison)

The records that were selected for qualitative review were divided in one or more of the following categories:

- Results obtained in a group of patients with different types of head and neck cancer (different in terms of primary location)
- Results obtained in a subgroup of patients with oropharyngeal cancer
- Results obtained in a subgroup of patients with cancer of the oral cavity
- Results obtained in a subgroup of patients with cancer of the hypopharynx/larynx
- Results for comparison of imaging techniques

In addition, records referring to guidelines pertinent to post-treatment surveillance of head and neck tumors were identified.

### Data collection process

We developed a data extraction sheet, pilot-tested it on ten randomly selected included studies, and used it accordingly (see Manuscript selection sheet). Data extraction was done by one author (S.V.H.) and reviewed by RH. Discrepancies were resolved in consensus.

### Extracted data items

The following quantitative data were extracted if available:

- Percentage of locoregional recurrences/metastases observed more than 6 months after treatment
- Average time of detection of locoregional recurrences/metastases (months after the end of treatment)
- Percentage of locoregional recurrences/metastases detected by surveillance imaging versus other methods

### Presentation of results

For clarity, results are divided in 4 categories:

1. Study selection (including PRISMA flowchart)
2. Overview of existing guidelines
3. Overview of included studies and qualitative results per category
4. Quantitative results

## Results

### Study selection

The following results were obtained:

- The PUBMED SEARCH: ((head and neck cancer[Title/Abstract]) AND (surveillance[Title/Abstract])) AND (imaging[Title/Abstract]) resulted in 133 hits. After exclusion of records obviously not covering the topic of the review, 91 records remained (Manuscript selection sheet, column B).
- The PUBMED SEARCH: ((head and neck cancer[Title/Abstract]) AND (surveillance[Title/Abstract])) AND (posttreatment[Title/Abstract]) resulted in 101 hits. After exclusion of records obviously not covering the topic of the review and duplicates, 69 records remained (Manuscript selection sheet, column C).
- References in NCCN guidelines: 6 records selected (Manuscript selection sheet, column D).
- The PUBMED SEARCH: ((head and neck cancer[Title/Abstract]) AND (follow-up[Title/Abstract])) AND (posttreatment[Title/Abstract]) resulted in 244 hits. After exclusion of records obviously not covering the topic of the review and duplicates, 74 records remained (Manuscript selection sheet, column E).
- The PUBMED SEARCH: ((head and neck cancer[Title/Abstract]) AND (surveillance[Title/Abstract])) AND (tomography[Title/Abstract]) resulted in 70 hits. After exclusion of records obviously not covering the topic of the review and duplicates, 50 records remained (Manuscript selection sheet, column F).
- The PUBMED SEARCH: (((cancer[Title/Abstract]) AND (surveillance[Title/Abstract])) AND (imaging[Title/Abstract])) AND (pharynx) resulted in 10 hits. After exclusion of records obviously not covering the topic of the review and duplicates, 3 records remained (Manuscript selection sheet, column G).
- The PUBMED SEARCH: (((cancer[Title/Abstract]) AND (surveillance[Title/Abstract])) AND (imaging[Title/Abstract])) AND (oral cavity[Title/Abstract]) resulted in 20 hits. After exclusion of records obviously not covering the topic of the review and duplicates, 8 records remained (Manuscript selection sheet, column H).
- The PUBMED SEARCH: (((cancer[Title/Abstract]) AND (surveillance[Title/Abstract])) AND (imaging[Title/Abstract])) AND (larynx[Title/Abstract]) resulted in 8 hits. After exclusion of records obviously not covering the topic of the review and duplicates, 2 records remained (Manuscript selection sheet, column I).
- The GOOGLE SCHOLAR SEARCH: “head and neck cancer posttreatment imaging surveillance”: resulted in 20,400 hits. The first 350 hits were checked. After exclusion of records obviously not covering the topic of the review and duplicates, 105 records remained (Manuscript selection sheet, column J, marked with “1”).
- The GOOGLE SCHOLAR SEARCH: “head and neck cancer posttreatment imaging follow-up”: resulted in 23,800 hits. The first 100 hits were checked. After exclusion of records obviously not covering the topic of the review and duplicates, 4 records remained (Manuscript selection sheet, column J, marked with “2”).
- The GOOGLE SCHOLAR SEARCH: “pharynx cancer imaging surveillance”: resulted in 15,000 hits. The first 100 hits were checked. After exclusion of records obviously not covering the topic of the review and duplicates, 8 records remained (Manuscript selection sheet, column J, marked with “3”).
- The GOOGLE SCHOLAR SEARCH: “oral cavity cancer imaging surveillance”: resulted in 46,700 hits. The first 100 hits were checked. After exclusion of records obviously not covering the topic of the review and duplicates, 12 records remained (Manuscript selection sheet, column J, marked with “4”).
- The GOOGLE SCHOLAR SEARCH: “larynx cancer imaging follow-up surveillance”: resulted in 25,300 hits. The first 100 hits were checked. After exclusion of records obviously not covering the topic of the review and duplicates, 16 records remained (Manuscript selection sheet, column J, marked with “5”).
- The GOOGLE SCHOLAR SEARCH: “head and neck cancer tomography surveillance”: resulted in 66,900 hits. The first 100 hits were checked. After exclusion of records obviously not covering the topic of the review and duplicates, 7 records remained (Manuscript selection sheet, column J, marked with “6”).
- The GOOGLE SCHOLAR SEARCH: “cited by”: for every article, a search through Google Scholar was conducted looking for (newer) articles that cite the initially found articles in their reference list (“cited by”). After exclusion of records obviously not covering the topic of the review and duplicates, 182 records remained (Manuscript selection sheet, column K).
- UPTODATE: Also was checked if the database "UpToDate" contained additional information or references. Eight new records were found (Manuscript selection sheet, column L).

In total, 521 records were identified through database search. In addition, 44 additional records were identified through other sources.

In total, 565 records were screened on basis of title and abstract. From these, 432 records were excluded. Reasons for exclusion were as follows: letters, review papers, editorials, case reports, studies on tumor types other than squamous cell carcinoma, tumors in locations other than nasopharynx, oropharynx, hypopharynx, larynx, or oral cavity, articles focusing on the first post-treatment scan (typically performed after 3–6 months), articles focusing on diagnosis, staging or treatment, articles failing to provide a comparison between systematic imaging-based surveillance and surveillance directed by symptoms and/or clinical/endoscopic exam, articles with methodological errors or missing information, articles in languages other than English, French, or Dutch, and articles published before 2000.

Thus, 133 (565 minus 432) records were promoted to the next step in the review process: assessment of full-text articles (Manuscript selection sheet, column M, marked with “x”). From these 133 articles, 85 were excluded for one or more of the above given reasons. So, 48 articles were selected for the final review (Manuscript selection sheet, column N, marked with “x”).

The process is summarized in Fig. 1.

### Overview of existing guidelines

An overview of existing international guidelines regarding surveillance imaging after the initial (3–6 months) post-treatment baseline imaging study are given in Table 1.

- As shown in Table 1, nine different guidelines were found during full text search of the selected articles. All guidelines support the use of initial (3–6 months) post-treatment imaging. However, subsequent imaging recommendations for asymptomatic patients span the spectrum from no routine imaging to regular periodic imaging.
- During our search, we found that many papers refer to the NCCN guidelines. In these guidelines, imaging after the first post-treatment scan is recommended only in locoregionally advanced disease and only if clinically indicated (symptomatic patient or suspect clinical finding). A possible exception to this general rule may be the post-treatment assessment of areas inaccessible to routine clinical examination (deep-seated anatomic locations or areas obscured by extensive treatment change). The NCCN guidelines also suggest that PET-CT may be the most sensitive imaging modality [8].
- While several guidelines [10, 12, 15, 16] follow the general NCCN preference for non-systematic symptom-directed imaging and/or clinical finding-directed imaging, some propose a clearly different approach. AWMF, for instance, recommends CT scan of the head and neck region every 6 months in the first 2 years after treatment and every 12 months for the third to fifth years [11]. The most intensive recommendations come from NI-RADS, which advocates (at minimum) annual surveillance imaging with more frequent studies for concerning or equivocal findings [17]. The NI-RADS approach makes follow-up strategies dependent on initial imaging findings. NI-RADS provides a structured reporting template and suggests classifying imaging findings in one of four categories: NI-RADS 1 (no evidence of recurrence), NI-RADS 2 (low suspicion, defined as ill-defined non-mass-like areas(s) of soft tissue with only mild differential enhancement or mild fluorine-18–2-fluoro-2-deoxy-D-glucose (FDG) uptake), NI-RADS 3 (high suspicion), and NI-RADS 4 (definite recurrence).

**Table 1:** Overview of existing international guidelines with regard to surveillance imaging after the initial (3–6 months) post-treatment baseline imaging study

Organization	Year	Guidelines
NCCN [8]	2022	• Imaging only in locoregionally advanced disease and only if clinically indicated • PET-CT may be the most sensitive imaging modality • If a PET/CT at 3 months post-treatment is negative, there are no data to support substantial benefit for further routine imaging in an asymptomatic patient with negative clinical exam • Routine annual imaging (repeat use of pretreatment imaging modality) may be indicated to visualize areas inaccessible to routine clinical examination (deep-seated anatomic locations or areas obscured by extensive treatment change)
eviCore 2.1 Clinical Guidelines [10]	2021	• No imaging surveillance after first post-treatment scan • Exceptions: in case of nasopharyngeal primary site or physical exam unable to visualize deep-seated primary site: annual CT or MRI for 3 years • In smokers: CT chest only if lung cancer screening criteria are met
AWMF (Germany) [11]	2012	• Imaging every 6 months in the first and second year, every 12 months in years 3–5
BAHNO (UK) [12]	2001	• None (symptom-directed only)
EHNS-ESMO-ESTRO [15]		• Imaging should be carried out if symptoms occur or in cases of abnormalities found at the clinical examination
ASCO [13]	2019	• Only if initial PET-CT shows possibly suspect lymph node
AHNS [16]	2016	• Consider in case of smoking history, nasopharyngeal primary, or tumor site inaccessible to clinical examination • Endorses NCCN guidelines
NI-RADS [17]	2018	• CT, MRI, or PET-CT every 3, 6, or 12 months depending on initial post-treatment imaging findings
ACS [14]	2016	• No definite recommendations

### Overview of included studies and summarized qualitative results per category

Tables 2, 3, 4, and Supplementary Tables 1 and 2 provide an overview of the studies that were selected for qualitative review in one of the following categories:

- Head and neck cancer in general (Table 2)
- Oropharyngeal cancer (Table 3)
- Cancer of the oral cavity (Table 4)
- Cancer of the hypopharynx/larynx (Supplementary Table 1)
- Results for comparison of imaging techniques (Supplementary Table 2)

**Table 2:** Post-treatment imaging of head and neck cancer beyond the first 3–6-month baseline imaging study. Overview of included studies evaluating the results obtained with routine imaging surveillance versus symptom-directed imaging and/or clinical finding-directed imaging

First author and year	Imaging technique(s)	Design/methods	Results/conclusion
**Imaging surveillance (at least once) after 3–6 months appears to be beneficial (in terms of lesion detection)**			
Alnefaie 2022 [25]	RX/CT (lung)	• Nationwide cross-sectional survey	• Pulmonary screening is believed to be very effective or somewhat effective
Ng 2020 [26]	mixed	• Markov decision process model	• For an infinite horizon policy, optimal scan intervals were between 10 and 18 months
Gore 2020 [27]	CT/PET-CT	• Retrospective study including 255 patients	• Imaging surveillance beyond the first posttreatment baseline study was critical for detecting clinically occult recurrent disease (36% of all recurrences)
Ng 2019 [28]	mixed	• Retrospective study including 1508 patients • Disease recurrences were classified as either clinically detected or imaging-detected	• 20% of patients with locoregional recurrence and 60% of distant recurrences did not present with a clinical finding and were detected by imaging • The yield of detecting a salvageable recurrence with routine imaging after 2 years in an asymptomatic patient with no adverse clinical findings is extremely low
Iovoli 2018 [29]	CT (lung)	• Retrospective study including 1114 patients	• Routine surveillance for HNSCC patients with lung CT imaging had value • Routine head and neck CT scans failed to identify any successfully salvaged patients
Jackowska 2018 [30]	mixed	• Retrospective study including 438 patients	• Routine imaging studies detected 25.9% of recurrences • 40.8% of recurrences were observed > 2 years after treatment
Meregaglia 2018 [31]	mixed	• Decision-analytic Markov model	• An intensive follow-up (comprising imaging tests twice a year in the first 2 years) appears cost-effective
Kim 2017 [32]	PET-CT	• Prospective study including 278 consecutive patients	• Posttreatment 18F-FDG PET/CT surveillance helped to properly detect recurrence and to predict survival • Median time to recurrence was 10 months
Kikuchi 2015 [33]	PET-CT	• Retrospective study including 158 patients	• 67% of tumor recurrences, including second primary cancers, were detected by routine surveillance with PET/ CT • PET/CT after the second scan (i.e., > 6–12 months after treatment) may be less effective
Jung 2014 [34]	CT/MRI/US	• Retrospective study including 520 patients	• 22.8% of recurrences were detected by screening imaging studies
Dunsky 2013 [35]	PET-CT	• Retrospective study including 123 patients	• 8% of PET-CT surveillance scans showed asymptomatic recurrence, at an average interval of 35.7 weeks posttreatment • Asymptomatic lesions were detected most frequently at distant sites
Kim 2013 [36]	PET-CT	• Retrospective study including 143 patients	• PET/CT is a useful tool for the detection of recurrent tumors at 3–6 and 12 months after curative treatment
Kostakoglu 2013 [37]	PET-CT	• Retrospective study including 99 patients	• FDG-PET/HRCT detected more disease recurrences or second primary cancers and did so earlier than CT or physical examination/endoscopy
McDermott 2013 [38]	PET-CT	• Retrospective study including 512 patients	• A single PET/CT with negative findings carries a NPV of 91%, which is not adequate to defer further radiologic surveillance • Two consecutive PET/CT examinations with negative findings within a 6-month period resulted in a NPV of 98%
Paidpally 2013 [39]	PET-CT	• Retrospective study including 134 patients	• PET/CT performed between 4 and 24 months after treatment adds value to clinical assessment
Beswick 2012 [40]	PET-CT	• Retrospective study including 388 patients	• For patients without clinical signs of recurrence, routine PET/CT surveillance beyond the first 24 months may be of limited value
Abgral 2009 [41]	PET-CT	• Prospective study including 91 patients	• PET/CT is more accurate than conventional follow-up physical examination alone • PET/CT could be proposed systematically at 12 months of the usual follow-up
Lee 2007 [42]	PET-CT	• Retrospective study including 159 patients	• For routine surveillance, the initial PET scan should be performed within 6 months after completion of treatment • The proper timing of next routine PET scan for subclinical patient with initial negative PET result might be 1 year after initial PET scan
**Imaging surveillance after 3-6 m appears to have no or only limited benefit**			
Iovoli 2018 [29]	CT	• Retrospective study including 534 patients	• Routine surveillance for HNSCC patients with lung CT imaging had value but routine head and neck CT scans failed to identify any successfully salvaged patients
Ho 2013 [43]	PET-CT	• Retrospective study including 1114 patients	• HNC patients with negative 3-month imaging appear to derive limited benefit from subsequent PET/CT surveillance
Sullivan 2010 [44]	CT	• Retrospective cohort study including 131 patients	• The utility of CT for surveillance may be limited
Saussez 2007 [45]	RX/US/CT	• Retrospective cohort study including 195 patients	• Systematic head and neck US and CT exams revealed recurrent cancers with poor efficiency and should be performed only after clinical suspicion of recurrence
**Imaging surveillance after 3-6 m is of uncertain benefit/other**			
Zhang 2011 [46]	PET-CT	• Retrospective cohort study including 62 patients	• A negative initial posttreatment PET/CT result may have the potential to identify patients who are at very low risk of recurrence • The HPV status may augment the predictive utility of an initial negative PET/CT result

**Table 3:** Post-treatment imaging of oropharyngeal cancer beyond the first 3–6-month baseline imaging study. Overview of included studies evaluating the results obtained with routine imaging surveillance versus symptom-directed imaging and/or clinical finding-directed imaging

First author and year	Imaging technique(s)	Design/methods	Results
**Imaging surveillance (at least once) after 3–6 months appears to be beneficial (in terms of lesion detection)**			
Nair 2022 [47]	CT	• Markov model based on data of 2159 patients	• Optimized, risk-stratified surveillance regimens consistently outperformed nonoptimized strategies. Best results were obtained with use of 6 post-treatment scans
Su 2018 [48]	PET-CT	• Retrospective cohort study including 33 patients	• In HPV+ patients, a large proportion of failures are asymptomatic distant metastases, which occur beyond 6 months following treatment completion, and are detected with whole body imaging alone
You 2018 [49]	PET-CT	• Retrospective cohort study including 149 patients	• PET-CT in the last 18 months of the 2-year posttreatment period impacted patient management
**Imaging surveillance after 3-6 m appears to have no or only limited benefit**			
Corpman 2018 [50]	PET-CT	• Retrospective study including 233 patients	• For HPV+ patients, surveillance PET-CTs frequently lead to unnecessary testing and rarely to meaningful disease salvage
Kangelaris 2010 [51]	MRI	• Retrospective study including 40 patients	• In oropharyngeal cancer patients who have been treated with chemoradiation, an imaging surveillance program utilizing MRI produces limited opportunity for successful salvage

**Table 4:** Post-treatment imaging of oral cavity cancer beyond the first 3–6-month baseline imaging study. Overview of included studies evaluating the results obtained with routine imaging surveillance compared to symptom-directed imaging and/or clinical finding-directed imaging

First author and year	Imaging technique(s)	Design/methods	Results
**Imaging surveillance (at least once) after 3–6 months appears to be beneficial (in terms of lesion detection)**			
Fukumoto 2021 [52]	CT/PET-CT	• Retrospective study including 324 patients	• It is desirable to perform PET/CT within 3–6 months and at 1 year after surgery and to consider CECT as an option in between PET/CT
Liu 2021 [53]	CT/MRI	• Retrospective study including 741 patients	• 19.7% of recurrences were found during routine imaging only • In late-stage and elderly patients, frequent head and neck CT/MRI scan was associated with a better prognosis
Ravanelli 2021 [54]	PET-CT	• Retrospective observational study including 87 patients	• Performing imaging studies every 6 months for 2 years changed the diagnostic/therapeutic strategy in about one-fifth of patients
Chi 2020 [55]	(?)	• Retrospective study including 83 patients	• 29.4% of recurrences were detected by serial imaging alone in asymptomatic patients; most of them (92.3%) occurred within the first 2 years
Lin 2017 [56]	PET-CT	• Retrospective study including 111 patients	• Scheduled periodic PET/CT surveillance is a valuable tool for early detection of recurrent lesion(s) in asymptomatic OSCC patients who bear risk factors for disease recurrence
Peisker 2017 [57]	RX/CT	• Retrospective study including 228 patients	• The results of this study suggest an intensified imaging follow-up within the first 2 years after surgery
Rivelli 2011 [58]	CT	• Retrospective study including 294 patients	• Routine CT for follow-up is indicated for detecting lymph node metastases as well as local recurrence
**Imaging surveillance after 3–6 months appears to have no or only limited benefit**			
Al-Shwaiheen 2014 [59]	MRI	• Retrospective study including 62 patients	• Routine MRI after 6 months may be unnecessary in patients without concurrent suspicious symptoms or exam findings
**Imaging surveillance after 3-6 m is of uncertain benefit/other**			
Krabbe 2009 [60]	PET-CT	• Prospective study including 48 patients	• PET is a suitable routine posttreatment surveillance tool and detects malignancy before clinical suggestion by the regular follow-up arises • The best timing of a systematic PET scan is between 3 and 6 months after treatment

#### Summary of results for head and neck cancer in general (Table 2)

- A large majority (18/25) of reports analyzed in this study suggest that systematic post-treatment surveillance imaging may be useful in terms of additional lesion detection when compared to a strategy where imaging is reserved for cases with suspect symptoms or clinical findings.
- In 11/17 studies with positive results, PET-CT was (predominantly or exclusively) used as imaging modality (65%). The average publication year of the studies with positive results was 2015. In 1/4 studies with negative results, PET-CT was (predominantly or exclusively) used as imaging modality (25%). The average publication year of the studies with positive results was 2013. In other words, there is a tendency toward better results in recently published studies using PET-CT as imaging modality.
- Most authors agree that imaging screening after 2 years is probably not effective. Several authors suggest performing surveillance imaging (mainly PET-CT) at 1 or 2 time points after the initial post-treatment scan. Suggested regimens are PET-CT exams at 12 months [41], 12 and 24 months [40], 9 months [33], 12 months [36], 12 months and 18–24 months [32], 18 months [42], 8 and 14 months [38], and 24 months [39].
- Of particular interest is the report of McDermott et al. [38]. These authors showed that a single PET-CT with negative findings carries a NPV of 91%, which the authors consider insufficient to defer further radiologic surveillance. On the other hand, two consecutive PET-CT examinations with negative findings within a 6-month period, resulted in an NPV of 98%, which could obviate further radiologic imaging in the absence of clinical signs of recurrence.

#### Summary of results for oropharyngeal cancer (Table 3)

Only five reports focused exclusively on oropharyngeal cancer (OPC). The results for oropharyngeal cancer are more mixed than those for the larger group of head and neck cancers. Among other factors, this may be related to the fact that this group contains both HPV-positive (HPV +) and HPV-negative (HPV −) OPCs, which have a quite distinct clinical behavior and prognosis.

#### Summary of results for cancer of the oral cavity (Table 4)

- As in the studies summarized in Table 2 (general group), most reports on oral cavity cancer Imaging surveillance (at least once) after 3–6 months appears to be beneficial in terms of lesion detection. Also, imaging surveillance after 2 years post-treatment appears to be less effective.
- When a free or pedicled flap reconstruction is required, a certain degree of distortion of the anatomical configuration of the residual hemitongue and floor of mouth is often produced, and clinical detection of recurrence may be difficult. It is not surprising that, in such cases, data show superior results with systematic imaging surveillance.

#### Summary of results for cancer of the hypopharynx/larynx (Supplementary Table 1)

- Only a limited number of studies is available for this body part. Submucosal recurrence appears to be an important issue, making imaging surveillance mandatory [61, 62].

#### Summary of results for comparison of imaging techniques (Supplementary Table 2)

- In this category, PET-CT appears to be the preferred modality for imaging surveillance [63–70]. Depending on the specific case (e.g., location of recurrence), MRI may be useful for confirmation and additional staging.

### Quantitative results

The quantitative results of the different studies are summarized in Table 5.

**Table 5:** Post-treatment imaging of head and neck cancer beyond the first 3–6-month baseline imaging study. Percentage of recurrences detected with surveillance imaging only, percentage recurrences after 6 months, and time to recurrence

**First author and year**	**Location**	**% Recurrence only detected with imaging**	**% Recurrence after 6 months**	**Median time to recurrence (months)**	**Time to recurrence: range**
Ng 2019 [26]	Mixed	38		12.5	(1–160)
Gore 2019 [27]	Mixed	36		11.4	
Jackowska 2018 [30]	Mixed	25.9	x		
Kim 2017 [32]	Mixed	47		10	(2–32)
Kikuchi 2015 [33]	Mixed	67	15	7.9	(1.3–78)
Jung 2014 [34]	Mixed	23	x		
Dunsky 2013 [35]	Mixed	20	64	9	(1–25)
Kim 2013 [36]	Mixed	86	33	11	(2–30)
Kostakoglu 2013 [37]	Mixed	84	50	6	(2.3–32)
Paidpally 2013 [39]	Mixed	21			
Beswick 2012 [40]	Mixed	66	55	6	(2–43)
Abgral 2009 [41]	Mixed	30	x	10.7	(1.3–20)
Lee 2007 [42]	Mixed	26	55		
Iovoli 2018 [29]	Mixed	50	x		
Ho 2013 [43]	Mixed	6	x	12	
Saussez 2007 [45]	Mixed	10			
**Average mixed**		**39.7**	**45.3**	**9.7**	
Su 2018 [48]	Oropharynx	84	91		
You 2018 [49]	Oropharynx		52.9		
Corpman 2018 [50]	Oropharynx	48		15	(7–24)
Kangelaris 2010 [51]	Oropharynx	50		15	(4–40)
**Average OP**		**60.7**	**72.0**	**15.0**	
Fukumoto 2021 [52]	Oral cavity			11.5	
Ravanelli 2021 [54]	Oral cavity	22			
Chi 2020 [55]	Oral cavity	29.4			
Lin 2017 [56]	Oral cavity	54		9.3	
Peisker 2017 [57]	Oral cavity	43.1			
Rivelli 2011 [58]	Oral cavity	16		21.7	(1–43)
Krabbe 2009 [60]	Oral cavity			7.2	
**Average OC**		**32.9**		**12.4**	
Marchi 2017 [62]	Hypopharynx/larynx			20	(8–60)
**Average all together**		**40.9**	**52.0**	**11.5**	

#### Summary of the quantitative results on recurrences (Table 5)

- For all records together, in summary, 40.9% locoregional recurrences and/or metastases were detected by routing post-treatment surveillance imaging and not by symptoms or clinical/endoscopic exam. Moreover, 52% of locoregional recurrences and/or metastases are detected after 6 months post-treatment, and the average time of detection of recurrent or metastatic disease is 11.5 months.
- Comparable results are obtained in the subcategories, except for oropharynx. For oropharynx, locoregional recurrences and/or metastases were detected by routine post-treatment surveillance imaging (and not by symptoms or clinical/endoscopic exam) in 60.7% versus 40.9% for the entire group. Moreover, the average time of detection of recurrent or metastatic disease was 15 months versus 11.5 months. In other words, imaging appears to be even more important in patients with treated oropharyngeal cancer (which may be related to the detection of asymptomatic distant metastases), and recurrences/metastases disease tend to occur later in time.

## Discussion

### Need for systematic imaging surveillance

Currently, there are no prospective randomized studies showing that systematic post-treatment surveillance imaging of patients with treated head and neck cancer is beneficial in terms of survival (with the exception of the first post-treatment study, see the “Introduction” section).

However, a large majority (18/25) of reports analyzed in this study suggest that systematic post-treatment surveillance imaging is useful in terms of additional lesion detection when compared to a strategy where imaging is reserved for cases with suspect symptoms or clinical findings. In 40.9% of cases, locoregional recurrences and/or metastases were detected by routine post-treatment surveillance imaging and not by symptoms or clinical/endoscopic exam. Thus, this systematic review provides strong arguments in favor of systematic post-treatment imaging surveillance beyond the first post-treatment scan.

This is not surprising for several reasons. First, in some anatomical locations (nasopharynx, subglottis, etc.), tumor detection and/or evaluation of submucosal spread may be difficult by clinical exam/direct inspection only. Second, tissue fibrosis, oedema, necrosis, and anatomic changes after radiotherapy and/or surgery can interfere with early detection of residual viable tumor or recurrence by the usual sequential physical and endoscopic examinations [71]. Third, a free or pedicled flap reconstruction may be required in patients with oral cavity cancer, often precluding the detection of submucosal relapse [54].

### Duration of systematic imaging surveillance

Another debated topic is the optimal length of post-treatment surveillance. Our results show that, on average, 52% of locoregional recurrences and/or metastases were detected after 6 months post-treatment, and the mean time of detection of recurrent or metastatic disease was 11.5 months. These numbers give strong arguments in favor of routine imaging surveillance during at least 1 (and preferably 2) year post-treatment. As suggested by others, routine surveillance imaging beyond the first 24 months may be of limited value and may not be cost effective [8, 40].

### The case of oropharyngeal cancer

The discussion about the usefulness of post-treatment surveillance in oropharyngeal cancer is complicated by the fact that HPV+ and HPV- OPCs can be considered as two different disease entities. The behavior of HPV+ poses an extra challenge for surveillance: on the one hand, HPV+ patients with OPC have better outcomes, a lower risk of recurrence and metastatic disease [72], and a longer overall survival, thus apparently making post-treatment surveillance less useful. On the other hand, recurrences in HPV+ OPC tend to occur later in time, and often correspond to distant metastases detected with whole body imaging only [48], thus providing theoretical arguments for prolonged screening with imaging. The peculiar behavior of oropharyngeal cancer was also observed in our study: in comparison with other head and neck cancers, oropharyngeal recurrences and/or metastases were detected later in time (15 months versus 11.5 months) and more exclusively by imaging (60.7%, versus 40.9% for the entire group).

Recently, promising results have been obtained with plasma circulating tumor HPV DNA for the surveillance of cancer recurrence in HPV+ oropharyngeal cancer [73, 74]. This type of tests could possibly be used for posttreatment surveillance in HPV+ patients, whereby the role of imaging could potentially be limited to confirmation of suspect cases.

### Imaging techniques for surveillance

Concerning the choice of imaging technique for post-treatment surveillance, we found better results with PET-CT when compared to CT or MRI. Treatment with radiotherapy and surgery causes inflammation, scarring, and tissue distortion, which can hinder the interpretation of anatomic imaging techniques such as CT and MRI. The metabolic information provided by 18F-FDG PET-CT allows it to serve as an effective tool for detecting recurrence, regional lymphatic spread, and distant metastases [39]. However, PET scanning is quite costly and may lead to additional diagnostic evaluation to rule out false positive results. As an alternative to PET-CT and depending on institutional preference, contrast-enhanced CT or MRI can be used for locoregional surveillance and low-dose CT for detection of metastatic lung disease [8]. MRI may also be useful for confirmation in case equivocal findings are found with PET-CT or for optimal assessment of specific anatomical areas (e.g., nasopharynx) [75].

### Overall conclusion

This systematic review focuses on the importance of systematic post-treatment imaging surveillance beyond the initial post-treatment scan in patients with head and neck cancer. While most current guidelines do not recommend post-treatment surveillance beyond the first post-treatment scan, we found strong arguments in favor of this approach.

Our data show that almost half of cases of locoregional recurrences and/or metastases were only detected by imaging (40.9%), and the mean time of detection of recurrent or metastatic disease (11.5 months) was well beyond the first post-treatment scan (3–6 months). As a result, we conclude that systematic imaging surveillance during at least 1 and possibly 2 years in the post-treatment period, with PET-CT being the preferred imaging modality, appears to be justified in cases of advanced head and neck cancer.

The further development of consensus guidelines regarding the surveillance of head and neck cancer by imaging methods would help to standardize the follow-up of these patients in the most effective way possible, with the goal of improving the eventual treatment outcome. This should ideally be done under the guidance of recognized organizations in the field.

### Supplementary Information

**Additional file 1: Supplementary Table 1.** Post-treatment imaging of cancer of the hypopharynx/larynx beyond the first 3–6-month baseline imaging study. Overview of included studies evaluating the results obtained with routine imaging surveillance compared to symptom- and/or clinical finding- directed imaging. **Supplementary Table 2.** Post-treatment imaging of head and neck cancer beyond the first 3–6-month baseline imaging study. Comparison of imaging techniques.

**Additional file 2: **Manuscript selection sheet.

## Data Availability

See Supplementary Table 3.

## Abbreviations

ACS: American Cancer Society
ASCO: American Society of Clinical Oncology
AWMF: Association of Scientific Medical Societies in Germany
HNSCC: Head and neck squamous cell carcinomas
HPV: Human papilloma virus
NCCN: National Comprehensive Cancer Network
NI-RADS: Neck imaging reporting and data system
NPV: Negative predictive value
OC: Oral cavity
OP: Oropharynx
OPC: Oropharyngeal cancer
RX: Radiography
