# Supplementary material for: Post-treatment surveillance imaging in head and neck cancer: a systematic review
Source: Insights Imaging. 2024 Feb 5;15:32. doi: 10.1186/s13244-023-01578-4 (PMC10844183; doi:10.1186/s13244-023-01578-4)
Supplement: Supplementary file 1 — Additional file 1: Supplementary Table 1. Post-treatment imaging of cancer of the hypopharynx/larynx beyond the first 3–6-month baseline imaging study. Overview of included studies evaluating the results obtained with routine imaging surveillance compared to symptom- and/or clinical finding- directed imaging. Supplementary Table 2. Post-treatment imaging of head and neck cancer beyond the first 3–6-month baseline imaging study. Comparison of imaging techniques. [file 13244_2023_1578_MOESM1_ESM.docx]

**Post-treatment surveillance imaging in head and neck cancer: a systematic review**

**ELECTRONIC SUPPLEMENTARY MATERIAL**

**Supplementary Table 1.**

Post-treatment imaging of cancer of the hypopharynx/larynx beyond the first 3–6-month baseline imaging study. Overview of included studies evaluating the results obtained with routine imaging surveillance compared to symptom- and/or clinical finding- directed imaging.

| **First author and year** | **Imaging technique(s)** | **Design / methods** | **Results** |
| --- | --- | --- | --- |
| **Imaging surveillance (at least once) after 3-6months appears to be beneficial (in terms of lesion detection)** | | | |
| Hermans 2000 (61) | CT | - Retrospective study including 66 patients | - In many patients, follow-up CT shows local failure earlier than does clinical examination alone |
| Marchi 2017 (62) | CT or MRI | - Retrospective study including 138 patients | - 43% of recurrent tumors were submucosal and therefore evident only at imaging |

**Supplementary Table 2**

Post-treatment imaging of head and neck cancer beyond the first 3–6-month baseline imaging study. Comparison of imaging techniques.

| **First author and year** | **Design / methods** | **Results** |
| --- | --- | --- |
| Morgan  2021  (63) | - Comparison of CT and PET-CT | - Compared with CT imaging, posttreatment imaging with PET was associated with improved survival in patients with advanced laryngeal carcinoma |
| House 2021  (64) | - Comparison of MRI and PET-CT | - PET-CT should be pursued as first-line surveillance tool for primary site recurrence following microvascular reconstruction where clinical evaluation is hindered by anatomical distortion - MRI can be utilized for confirmation of positive PET-CT findings |
| Breik  2020  (65) | - Comparison of MRI and PET-CT | - PET-CT is preferable to MRI for identifying post treatment locoregional and distant treatment failure |
| Suenaga  2016  (66) | - Comparison of PET, non-contrast-enhanced CT, and contrast-enhanced CT | - FDG-PET/CT was a more accurate surveillance tool than ceCT. The added value of ceCT at FDGPET/ CT is minimal |
| Ichpujani  2014  (67) | - Comparison of PET- CT and contrast-enhanced CT | - PET-CT not only is superior to conventional CECT in the detection of recurrence at locoregional and lymph node sites, but also plays a role in the detection of distant metastases and second primary tumors |
| Ghanooni  2011  (68) | - Comparison of MRI and PET-CT | - Performance of PET-CT and MRI are similar except for a higher sensitivity of PET-CT at 4 months |
| Wang 2008 (69) | - Comparison of PET- CT and contrast-enhanced CT | - PET had better accuracy than CT, both for tumor recurrence and lymph node metastases |
| Fakhry  2007  (70) | - Comparison of PET and PET-CT | - Combined PET/CT is more accurate than PET alone for detection of recurrent HNSCC |
